# Facts and Remarks on the Sensibility to Caloric

**Published:** 1840-11

**Authors:** Thomas Townsend

**Affiliations:** Wheeling, Va.


*August*, 1840.

---

### Art. III.—*Facts and Remarks on the Sensibility to Caloric.* By Thomas Townsend, M. D., of Wheeling Va.

Darwin, in the first volume of his Zoonomia, Sec. XIV., after speaking of the universal distribution of heat through all bodies in nature, suggests the idea that there may be, and probably is, a separate set of nerves from those of touch to distinguish heat. "There is no circumstance," says he, "of more consequence in the animal economy than a due proportion of this fluid of heat; the digestion of our nutriment in the stomach and bowels, and the proper qualities of all our secreted fluids being produced or prepared partly by animal and partly by chemical processes, depend much on the quantity of heat; the excess of which or its deficiency alike gives us pain, and induces us to avoid the circumstances that occasion them. And in this the perception of heat essentially differs from the perception of the sense of touch, as we receive pain from too great pressure of solid bodies, but none from the absence of it. It is hence probable, that nature has provided us with a separate set of nerves for the perception of this fluid, which anatomists have not yet attended to."

In confirmation of this supposition he says: "If we look at a hot fire, we experience no pain in the optic nerve, though the heat along with the light must be concentrated upon it, nor does warm oil or warm water poured into the ear give pain to that organ; and hence their organs do not perceive small excesses or deficiencies of heat." He thinks that heat has no greater analogy to the solidity or figure of bodies than to their color or vibrations, and that there is not sufficient reason for our ascribing the perception of heat and cold to the sense of touch, to which it has generally been attributed.

"There is another circumstance," he says, "wonld induce us to believe, that the perception of heat and cold do not belong to the organ of touch; since the teeth, which are the least adapted for the perception of solidity or figure, are the most sensible to heat or cold; hence we are forewarned from swallowing those materials, whose degree of heat or coldness would injure our stomachs. * * * The lungs are not sensible to heat and cold."

Darwin then gives the following extract of a letter of his relation Dr. R. W. Darwin, of Shrewberry, when a student at Edinburgh: "I made an experiment yesterday in our hospital, which much favors your opinion, that the sensations of heat and touch depend on different sets of nerves. A man who had lately recovered from fever, and was still weak was seized with violent cramps in his legs and feet; which were removed by opiates, except that one of his feet remained insensible. Mr. Ewart pricked him with a pin in five or six places, and the patient declared that he did not feel it in the least, nor was he sensible of a very smart pinch. I then held a red hot poker at some distance, and brought it gradually nearer, till it came within three inches, when he asserted that he felt it quite distinctly."

In the year 1812, when I was a student with Dr. M'Namee, of Vincennes, Indiana, Judge Vanderburg of that place was attacked with general palsy which came on slowly, but at length he lost the power of his limbs and became confined to bed. Dr. M'Namee was his attending physician, and Dr. Scull, formerly of Pittsburgh, consulting physician. During the time they were using electricity as one of their remedial agents, I solicited them to make Darwin's experiment, for the purpose of ascertaining whether the Judge could distinguish heat, after having lost the sense of touch together with the power of muscular motion. To this they assented.

The Judge lay in a room, the door of which, was opposite to the foot of his bed. He was not informed of the intended experiment. For the experiment the bedclothes were thrown from his feet and legs upon his breast so high that he could not see as low as the top of the door, and consequently could not see the entrance of the servant who brought in the fire. The feet and legs being thus uncovered were first pricked with a pin, and then pinched as hard as was consistent with humanity, and he declared he felt nothing. Then a signal was given and a servant entered with a shovel of hot coals. This was brought within twelve or fifteen inches of his feet and legs, when he immediately exclaimed, What, what! What is that, that is so warm?

This experiment clearly showed that the power of perceiving heat remained unimpaired, notwithstanding there was an entire loss of the sense of touch or feeling, and the power of muscular motion or volition, and went to confirm the opinion of Darwin, that there are separate sets of nerves for these separate functions.

Since the time of Darwin, and since the experiment of Drs. M'Namee and Scull, Sir Charles Bell has, by dissections and experiments, most satisfactorily demonstrated the existence of separate sets of nerves for separate and specific functions besides hearing, seeing, and smelling. He has shown that one set ministers to the function of what he calls common sensation, that is, touch. Another to the function of muscular motion, that is, volition, and another to the function of associating the apparatus of respiration with all other parts of the body. This additional knowledge on the physiology of the nervous system affords strong analogy in favor of the supposition that there may be, and probably is, a separate set of nerves for distinguishing heat.

In August, 1835, James Howell, of Wheeling, Virginia, was attacked with cholera morbus six or seven miles from town, from which he soon recovered and returned to his residence. A day or two after his return he was suddenly seized with a paralysis. When I visited him he complained of feeling "dead," as he expressed it. Mr. Howell had not lost all muscular power; he could move his hands and arms a little, and make some ineffectual efforts to turn himself in bed. He soon recovered from this and was able to walk. But he made the discovery that he could not feel any heat. He said that he "felt all the time as cold as ice"—that when he came to the fire he could not tell by his sense of feeling when he was warm, or whether he was burning. At a time when he said his feet and legs felt "as cold as ice," I put my hand on them and they felt to me as warm as the flesh of ordinary persons.

To-day, July 27, 1840, Mr. Howell called on me, when I read him the foregoing paragraph, and he said it was strictly true. He moreover stated, that from the back part of his head down the whole length of his spine, he feels all the time cold, and from the calf of his legs down he feels no heat. I felt his skin again, and it was to my hand as warm as that of any other person. He farther stated, that from 1835 to the present time, he has had great weakness of his hands and arms, and frequently can scarcely use his knife and fork in eating, and that he is subject to frequent cramps in his legs.

This case is the opposite of that of Judge Vanderburg, of Vincennes, who retained the power of distinguishing heat after that of muscular motion and of touch or feeling was lost. Mr. Howell retains to a considerable extent the power of muscular motion and feeling, while the ability to distinguish or perceive heat is lost.

I think that physiological facts like these, as suitable cases may present themselves, ought to be recorded for the purpose of leading to farther observations.

The truth of Mr. Howell's case depends on his own veracity, and, so far as I know, there is no one in Wheeling who will doubt it. But I have had no opportunity to make any experiment on him during sleep for the purpose of testing it.

